# Inpatient antibiotic prescribing patterns using the World Health Organization (WHO) Access Watch and Reserve (AWaRe) classification in Okinawa, Japan: A point-prevalence survey

**DOI:** 10.1017/ash.2022.263

**Published:** 2022-09-14

**Authors:** Payal K. Patel, Naoyuki Satoh, Masashi Narita, Yoshiaki Cho, Yusuke Oshiro, Tomoharu Suzuki, Karen E. Fowler, M. Todd Greene, Yasuharu Tokuda, Keith S. Kaye

**Affiliations:** 1 Division of Infectious Diseases, Veterans’ Affairs Ann Arbor Healthcare System and University of Michigan, Ann Arbor, Michigan, United States; 2 Division of Hospital Medicine, Heartlife Hospital, Okinawa, Japan; 3 Division of Infectious Diseases, Okinawa Chubu Hospital, Okinawa, Japan; 4 Division of Pediatric Infectious Diseases, Okinawa Prefectural Nanbu Medical Center and Children’s Medical Center, Okinawa, Japan; 5 Division of Hospital Medicine, Urasoe General Hospital, Okinawa, Japan; 6 Center for Clinical Management Research, VA Ann Arbor Healthcare System, Ann Arbor, Michigan, United States; 7 Muribushi Okinawa Center for Teaching Hospitals, Okinawa, Japan; 8 Division of Infectious Diseases, Robert Wood Johnson Medical School, New Brunswick, New Jersey, United States

## Abstract

Using point-prevalence methodology and the World Health Organization (WHO) Access, Watch, and Reserve Classification, we measured antibiotic use in 5 hospitals in Okinawa, Japan, on October 1, 2020. Overall, 29% of patients were prescribed an antibiotic on the survey date and the 3 most used antibiotics in the “Watch” category were cefazolin, ampicillin-sulbactam, and ampicillin.

Recently, a nationwide push in Japan has spurred action against antimicrobial resistance, including establishment of a national action plan.^1^ Although the prevalence of antibiotic stewardship programs (ASPs) has increased in Japan, published data on the uptake of ASPs in the country are limited. To better inform nationwide efforts on antibiotic use, we sought to understand inpatient antibiotic use in the prefecture of Okinawa, Japan, where there has been little published in the realm of antimicrobial stewardship.^2^ Point-prevalence studies of antibiotic use have been used internationally to provide regional data on antibiotic use.^3–5^ The World Health Organization (WHO) Access, Watch and Reserve (AWaRe) Classification is a tool developed in 2019 that can compare antibiotic use from different geographic regions. The AWaRe criteria were intended to improve international antibiotic monitoring and use, with an emphasis on potential for antimicrobial resistance. “Access” antibiotics are common frontline antibiotics, “Watch” antibiotics are high-priority antibiotics with toxicity or resistance concerns, and “Reserve” antibiotics are last-line treatments for multidrug-resistant infections. We hypothesized that Access and Watch antibiotics would comprise most antibiotics used in this group of hospitals.

## Methods

We evaluated the proportion of total eligible hospitalized patients who received antibiotics on the assessment date. We also categorized antibiotics according to indication, drug class, and AWaRe classification.

A point-prevalence study was conducted in 5 hospitals in Okinawa, Japan, on October 1, 2020. These hospitals are community hospitals, with bed sizes ranging from 308 to 559 beds. Each hospital has 1 physician antimicrobial stewardship champion, and 3 of these hospitals have an ID-trained physician as the antimicrobial stewardship champion. All 5 hospitals also had a pharmacist and microbiologist on the antimicrobial stewardship team. All antimicrobial stewardship physician champions had supported, dedicated effort from their hospital to conduct antimicrobial stewardship. All inpatients who were hospitalized on October 1, 2020, the day of data collection, were included. A site physician champion at each site conducted chart reviews of all inpatients receiving intravenous antibiotics. A pilot point-prevalence study was conducted by the lead Japanese author (N.S.) at a single hospital to help optimize the data collection sheet and process. Type of antibiotic, reason for use, duration, and microbiologic data were collected. Information on whether the antibiotic was being used empirically or directed at a specific pathogen was also collected.

Descriptive statistics were used to estimate the point prevalence of antibiotic use and to derive the distributions of AWaRe classifications and drug class. All statistical analyses were conducted using Stata MP version 14.1 software (StataCorp, College Station, TX).

## Results

Across the 5 hospitals, 1,728 unique patients were included. On the assessment date, 504 (29%) received at least 1 antibiotic. In total, 559 antibiotics were used for these 504 patients. Of those patients prescribed an antibiotic, 451 (89.5%) received 1 antibiotic, 51 (10.1%) received 2 antibiotics, and 2 (0.4%) received 3 antibiotics. Overall, 123 antibiotics (22.0%) prescribed across 114 patients were for prophylaxis and 436 antibiotics (78.0%) prescribed across 390 patients were for treatment. Of the 390 patients who received antibiotics, 385 (98.7%) had a documented infection source. The most common treatment indications for antibiotic use were pneumonia (n = 93, 23.8%), urinary tract infection (n = 76, 19.5%), and intra-abdominal infection (n = 69, 17.7%).

**Fig. 1.**
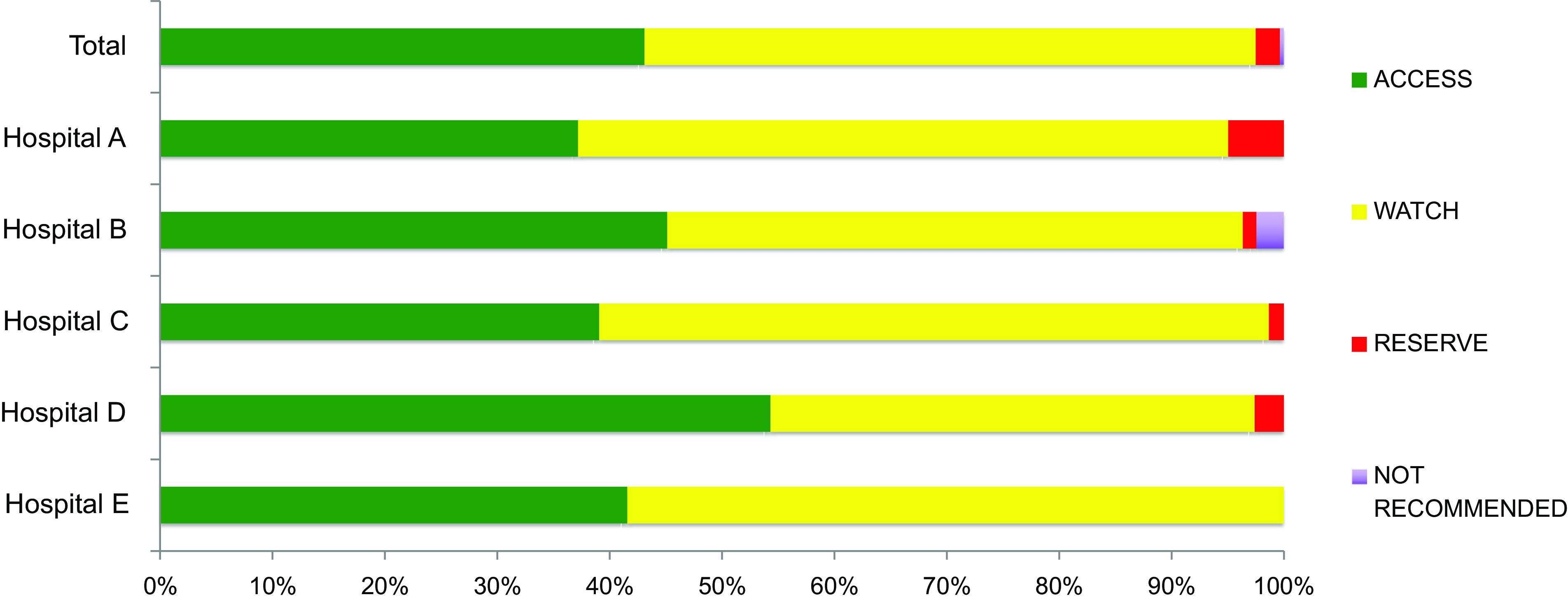
WHO AWaRe classification antibiotics distribution.

**Fig. 2.**
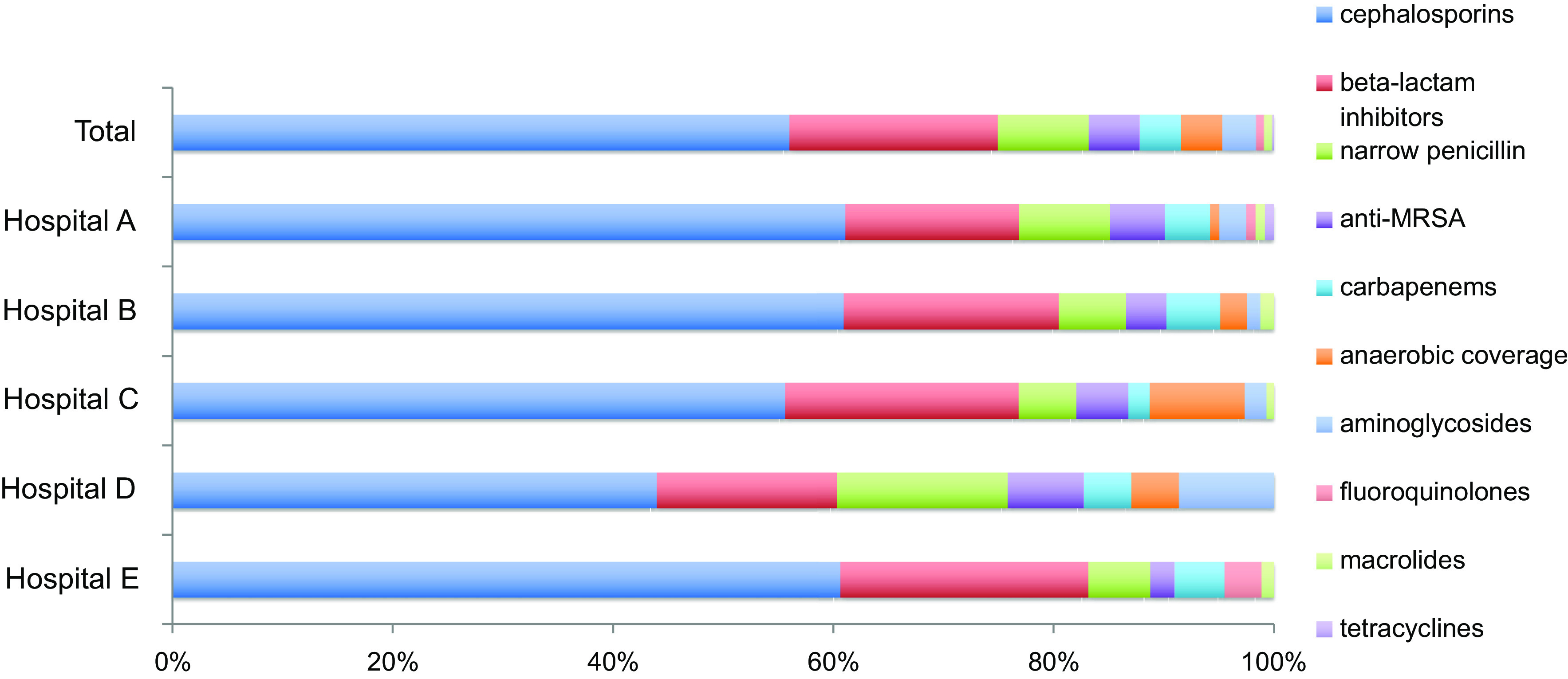
Antibiotic class distribution.

Overall, 241 (43.1%) of all 559 antibiotics were categorized as Access drugs, 304 (54.4%) were Watch drugs, and 12 (2.1%) were Reserve drugs. Only 2 (0.4%) of the antibiotics prescribed were in the “Not Recommended” category (Fig. 1). Cephalosporins were the most prescribed antibiotic class (n = 313, 56.0%), followed by ß-lactam/ß-lactamase inhibitors (n = 106, 19.0%) and narrow-spectrum penicillins (eg, penicillin G; n = 46, 8.2%) (Fig. 2).

Cefazolin (n = 92, 38.2%) and ampicillin-sulbactam (n = 67, 27.8%) were the most used antibiotics in the Access category. Cefmetazole (n = 94, 30.9%) and ceftriaxone (n = 52, 17.1%) were the most used antibiotics in the Watch category. The antibiotics most used in the Reserve category were aztreonam (n = 6, 50%) and daptomycin (n = 5, 42%).

The classification of directed versus empiric therapy was available for 488 (87.3%) of the antibiotics prescribed. When comparing directed therapy to empiric therapy, categorizations in the AWaRe categories were similar to overall findings. Among agents used for directed therapy, 60 (38.2%) were in the Access category, 89 (56.7%) were in the Watch category, and 8 (5.1%) were in the Reserve category. In the empiric therapy category, 134 (40.2%) were in the Access category, 193 (58%) were in the Watch category, and 4 (1.2%) were in the Reserve category.

In conclusion, 29% of inpatients in these 5 Okinawan hospitals were prescribed an antibiotic on the survey date. Regional data on antibiotic use varies, but this number is lower than has been reported in many international studies; however, it is similar to previous published work from Japan.^3,4,6,7^ Although national and regional information is scarce regarding the composition of stewardship programs, study hospitals all had physician stewardship champions, often infectious diseases-trained, with supported effort for stewardship activity. In addition, several programs had pharmacy and microbiology support. These characteristics might not be common in Japan and are very uncommon internationally.

In the Access category, the 3 most used antibiotics were cefazolin, ampicillin-sulbactam and ampicillin. In the Watch category, the 3 most used antibiotics were all cephalosporins (ie, cefmetazole, ceftriaxone, and cefotaxime). In the Reserve category, the most used antibiotic was aztreonam. Japanese Respiratory Society Guidelines for management of pneumonia recommend ampicillin-sulbactam and ceftriaxone and cefotaxime for nonsevere healthcare-associated pneumonia treatment and ampicillin-sulbactam and ceftriaxone as first-line therapy for admitted ward patients for nonsevere community-acquired pneumonia. Given that the most common indication for antibiotics in our study was pneumonia, concordance with national guidelines provides some insight into antibiotic use in this study. Moreover, 22% of all antibiotics used were also for prophylaxis, and this could explain some of the cefazolin use, the most used Access antibiotic in our study. A previous national shortage of cefazolin in Japan in 2019 led to a significant increase in third-generation cephalosporins, especially ceftriaxone.^8^ Due to multifaceted issues involving industrial complexities and supply chain fragility, the shortage took 10–11 months to resolve. Drug shortages can critically affect a prescriber’s choice of antibiotic and have been shown to influence appropriateness of antibiotic use in Japan and supply of other antibiotics.

The strengths of this work are that a multicenter point-prevalence survey of antibiotic use has not been done in this region of Japan and could inform future work in antibiotic stewardship. This study also had several limitations. Point-prevalence surveys are restricted to a single point in time, so variation of trends in antimicrobial use or seasonality may not be adequately represented. In addition, appropriateness of antimicrobial agents was not assessed. Given that we studied only 5 hospitals, generalizability to other institutions is limited. Additionally, patient-level data were not collected.

The most used antibiotics in this study fall under the WHO AWaRe Watch classification. Antibiotics in this classification may be more likely to cause resistance compared to Access antibiotics. Recent international work has shown that there is significant variability in how different countries use antibiotics when compared to using the AWaRe classificiation.^9,10^ Focusing on antimicrobial stewardship strategies (eg, selection of antibiotics for national or institutional guideline recommendations) that consider risk of antimicrobial resistance could be effective and beneficial. The impacts of such strategies could be assessed using repeated point-prevalence studies and the WHO AWaRe classification. Further work to understand how antibiotics are selected at Japanese hospitals would support the idea that using the AWaRe framework when considering institutional or national antimicrobial stewardship guidelines could affect the choice of antibiotic.

## References

[1] Kusama Y , Tsuzuki S , Muraki Y , Koizumi R , Ishikane M , Ohmagari N. The effects of Japan’s National Action Plan on Antimicrobial Resistance on antimicrobial use. Int J Infect Dis 2021;103:154–156. 33227519 10.1016/j.ijid.2020.11.158

[2] Taniguchi T , Tsuha S , Shiiki S , Narita M. Gram-stain–based antimicrobial selection reduces cost and overuse compared with Japanese guidelines. BMC Infect Dis 2015;15:458. 26503359 10.1186/s12879-015-1203-6 PMC4623896

[3] Thu TA , Rahman M , Coffin S , Harun-Or-Rashid M , Sakamoto J , Hung NV. Antibiotic use in Vietnamese hospitals: a multicenter point-prevalence study. Am J Infect Control 2012;40:840–844. 22341530 10.1016/j.ajic.2011.10.020

[4] Komagamine J , Yabuki T , Kobayashi M , Okabe T. Prevalence of antimicrobial use and active healthcare-associated infections in acute-care hospitals: a multicentre prevalence survey in Japan. BMJ Open 2019;9:e027604. 10.1136/bmjopen-2018-027604 PMC6609065 31256027

[5] Morioka H , Iguchi M , Tetsuka N , et al. Five-year point prevalence survey of healthcare-associated infections and antimicrobial use in a Japanese university hospital. Infect Prev Pract 2021;3:100151. 34647007 10.1016/j.infpip.2021.100151 PMC8498696

[6] Al Matar M , Enani M , Binsaleh G , et al. Point-prevalence survey of antibiotic use in 26 Saudi hospitals in 2016. J Infect Public Health 2019;12:77–82. 30270148 10.1016/j.jiph.2018.09.003

[7] Porto APM , Goossens H , Versporten A , Costa SF. Global point-prevalence survey of antimicrobial consumption in Brazilian hospitals. J Hosp Infect 2020;104:165–171. 31678430 10.1016/j.jhin.2019.10.016

[8] Honda H , Murakami S , Tokuda Y , Tagashira Y , Takamatsu A. Critical national shortage of cefazolin in Japan: management strategies. Clin Infect Dis 2020;71:1783–1789. 32133482 10.1093/cid/ciaa216

[9] Hsia Y , Lee BR , Versporten A , et al. Use of the WHO Access, Watch, and Reserve classification to define patterns of hospital antibiotic use (AWaRe): an analysis of paediatric survey data from 56 countries. Lancet Glob Health 2019;7:e861–e871. 31200888 10.1016/S2214-109X(19)30071-3

[10] Pauwels I , Versporten A , Drapier N , Vlieghe E , Goossens H. Hospital antibiotic prescribing patterns in adult patients according to the WHO Access, Watch and Reserve classification (AWaRe): results from a worldwide point-prevalence survey in 69 countries. J Antimicrob Chemother 2021;76:1614–1624. 33822971 10.1093/jac/dkab050 PMC8120336

